# Insecticide-induced leg loss does not eliminate biting and reproduction in *Anopheles gambiae* mosquitoes

**DOI:** 10.1038/srep46674

**Published:** 2017-04-25

**Authors:** Alison T. Isaacs, Amy Lynd, Martin J. Donnelly

**Affiliations:** 1 Department of Vector Biology, Liverpool School of Tropical Medicine, Liverpool, UK; 2 Malaria Programme, Wellcome Trust Sanger Institute, Cambridge, UK

## Abstract

Recent successes in malaria control have been largely attributable to the deployment of insecticide-based vector control tools such as bed nets and indoor residual spraying. Pyrethroid-treated bed nets are acutely neurotoxic to mosquitoes, inducing symptoms such as loss of coordination, paralysis, and violent spasms. One result of pyrethroid exposure often seen in laboratory tests is mosquito leg loss, a condition that has thus far been assumed to equate to mortality, as females are not expected to blood feed. However, whilst limb loss is unlikely to be adaptive, females with missing limbs may play a role in the propagation of both their species and pathogens. To test the hypothesis that leg loss inhibits mosquitoes from biting and reproducing, mosquitoes with one, two, or six legs were evaluated for their success in feeding upon a human. These experiments demonstrated that insecticide-induced leg loss had no significant effect upon blood feeding or egg laying success. We conclude that studies of pyrethroid efficacy should not discount mosquitoes that survive insecticide exposure with fewer than six legs, as they may still be capable of biting humans, reproducing, and contributing to malaria transmission.

Despite decades of public investment in vector control campaigns and industrial investment in pesticide research, the mosquito remains the deadliest animal in the world^1^. The bite of a mosquito can transmit a variety of human disease agents, including malaria parasites, filarial worms, and viruses such as dengue, chikungunya, and zika. The most widely deployed mosquito control tools are insecticide-based, functioning to either repel or kill mosquitoes within a home. One class of insecticides, pyrethroids, is employed both domestically, on bed nets and for indoor residual spraying, and in agriculture. These neurotoxic substances act upon a wide range of insects, inducing a “knockdown effect” characterized by a loss of coordination, spasms, tremors, and paralysis^2^. In mosquitoes, pyrethroid exposure frequently leads to the loss of one or more legs (Supplementary Video 1)^3^,^4^,^5^. Non-target invertebrates such as the stonefly exhibit similar symptoms following pyrethroid exposure, including arched backs and random leg movement^6^. In the diamondback moth, leg loss has been hypothesized to be an adaptive response, allowing the insect to reduce insecticide poisoning^7^. In mosquitoes, however, leg loss has been observed in pyrethroid-exposed mosquitoes that had no direct insecticide-leg contact, suggesting that leg fracture is a result of strained leg flexion^8^.

WHO guidelines for testing of long-lasting insecticidal nets dictate that mosquitoes should be classed as moribund if they cannot stand (e.g. have 1 or 2 legs)^9^. Moribund mosquitoes are placed in the same category as dead mosquitoes, and are therefore considered knocked down at 60 minutes post-exposure, and dead at 24 hours post-exposure. Some studies of pyrethroid efficacy have attempted to refine the classification of post-exposure outcomes by labelling mosquitoes that have suffered leg loss as either dead, or within a “functional mortality” category^10^,^11^,^12^. While the precise categorization of amputee mosquitoes varies among publications, it is generally assumed that they would not survive to bite again, or transmit disease.

The objective of this study was to test the hypothesis that leg loss prevents mosquitoes from feeding upon humans. To do so, the blood feeding and egg laying success of *Anopheles gambiae* mosquitoes that had lost legs either through amputation or pyrethroid exposure was compared to six-legged controls.

## Results

### Impact of leg loss via amputation upon blood feeding and egg laying

**Figure 1:**
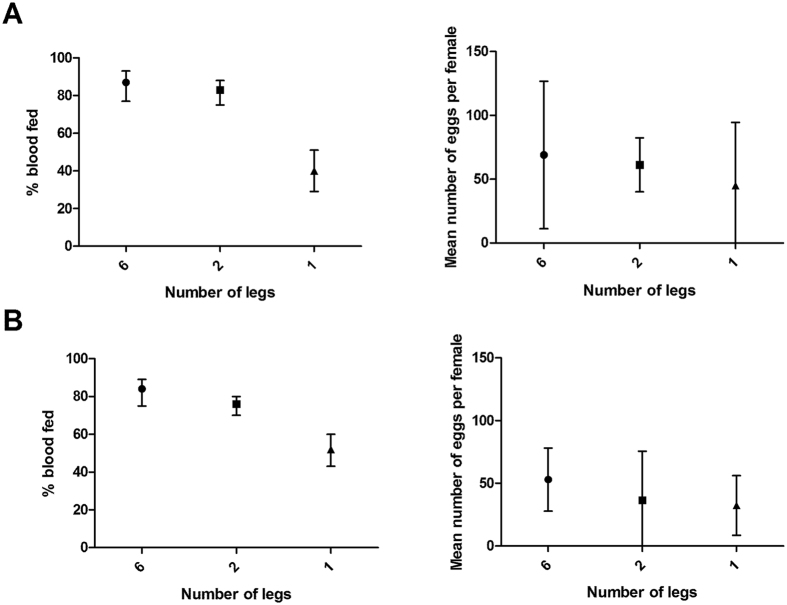
Blood feeding success and egg production in mosquitoes with 6, 2, or 1 leg. Mean values and 95% confidence intervals are shown. (**A**) Mosquito legs amputated mechanically. Data were compiled from three experimental replicates. (**B**) Mosquito leg loss induced by insecticide-exposure. Data were compiled from five experimental replicates.

**Figure 2:**
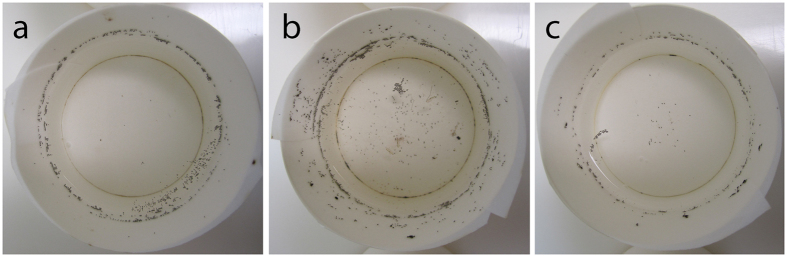
Eggs laid by cages of mosquitoes with (**a**) 6 legs, (**b**) 2 legs, (**c**) 1 leg. In contrast to controls, eggs from amputee mosquitoes were found widely scattered along the filter paper lining the sides of the cup and sunken on the bottom of the cup.

In three independent experiments, each performed using a different generation of mosquitoes, females in all control and amputee cages (6 legs, 2 legs, 1 leg) were able to successfully blood feed and lay eggs (Fig. 1A, Supplementary Video 2). The blood feeding success, summarised across all experiments, of 6- and 2-legged females was strikingly similar, with 87% (95% CI [77, 93]) and 83% (95% CI [75, 88]) fed, respectively. One-legged females displayed a reduced but appreciable blood feeding success, with 40% (95% CI [29, 51]) fed. In a generalized linear mixed model (GLMM) with replicate added as a random effect, there was a significant effect of leg number upon blood feeding success (ANOVA model test p = 0.004). Mortality, as measured 24 hours after amputation and post-blood feeding, was low (0–3 individuals per treatment), and likely due in part to accidental injury during hand feeding. Furthermore, blood-fed females displaying all 2-legged and 1-legged amputation patterns were recovered. The number of eggs per blood-fed female did not differ between treatments (GLMM with replicate as a random effect, ANOVA model test p = 0.152), with 6-, 2-, and 1-legged females producing an average of 69, 61, and 45 eggs per female, respectively. However, egg cups from amputee cages were consistently seen to have a different spatial distribution of eggs, including both sunken eggs and a wide scattering of eggs upon the filter paper (Fig. 2). During egg counting, newly hatched larvae were observed on all egg papers.

### Impact of leg loss via pyrethroid exposure upon blood feeding and egg laying

In five independent experiments performed using four different generations of mosquitoes, females that survived insecticide exposure with only one or two legs were observed to blood feed and lay eggs (Fig. 1B). The blood feeding success rates, summarised across all experiments, of 6-, 2-, and 1-legged females were similar to those of amputee experiments, with 84% (95% CI [75, 89]), 76% (95% CI [70, 80]), and 52% (95% CI [43, 60]) fed, respectively. This trend resembles that of the amputee experiments and in a GLMM of blood feeding success as a function of leg number which controlled for replicate, inclusion of the method of amputation (dissection or insecticide exposure) did not result in a significant improvement in model fit (p > 0.05). For the insecticide-exposed treatment group there were high levels of inter-replicate variation. In a GLMM with replicate added as a random effect, leg number was not a significant predictor (ANOVA model test, p = 0.078). In these experiments, females were placed individually into egg laying vials, which allowed for measurement of both egg laying success and egg batch size. No effect of leg number was detected in egg laying success, with 6-, 2-, and 1-legged females displaying 64% (95% CI [53, 73], 68% (95% CI [60, 75], and 50% (95% CI [36, 64] success in laying eggs, respectively (simple logistic regression, ANOVA model test p = 0.303). A logistic regression model is included here for illustration, as mixed-effect models did not converge because some replicates were missing data for some leg number by egg laying success combinations. Inducing leg loss by insecticide exposure is an imprecise art. Mean egg batch size was comparable between treatment groups, with 6-, 2-, and 1-legged females yielding 87, 83, and 73 eggs/female, respectively (GLMM, ANOVA model test, p = 0.498).

## Discussion

In a first set of experiments, mechanical amputation was used to test the effect of leg loss upon blood feeding success in the absence of insecticide exposure. Exposure to permethrin in a cone assay then measured the cumulative impact of leg loss and pyrethroid exposure upon blood feeding, yielding remarkably similar results. Mosquitoes that survived insecticide exposure with only one or two legs displayed no significant difference in blood feeding success or egg production compared to six-legged controls. While not statistically significant, a lower rate of blood feeding success was observed in 1-legged insecticide-exposed mosquitoes, similar to that which was seen in 1-legged amputee mosquitoes. With one leg, mosquitoes maintain their ability to fly, land on a vertical surface, and hang underneath a horizontal one. While their egg laying behaviour appears to be altered, the presence of larvae indicated that fertile eggs were laid.

The wide scattering of eggs and presence of sunken eggs in the oviposition cups of 1- and 2-legged mosquitoes suggests that laying behaviour was perturbed by leg amputation. In the laboratory environment, *Anopheles* mosquitoes have been seen to lay eggs whilst alighted on the surface of the water or perched at its edge^13^,^14^,^15^. While oviposition has rarely been observed in field conditions, the ability of floating sticky traps to capture gravid *Anopheles* suggests that direct contact with the water surface is a component of this behaviour^16^. In addition, several 1- and 2-legged females were seen either dead or apparently trapped on the water surface at the time of egg counting. The stress of entrapment may have induced sub-optimal oviposition, a behaviour previously associated with chemical and mechanical stresses^17^,^18^.

While the lifetime fitness of 1 or 2-legged mosquitoes is not known, mortality following leg amputation was minimal, suggesting that the wound-healing response is highly efficient in preventing a loss of haemolymph and dehydration. Laboratory mosquitoes encounter pathogens in the insectary environment, but are still likely to have a longer lifespan than field mosquitoes because they do not expend energy searching for food or avoiding predators. Performing leg counts on field-collected mosquitoes would provide an indication whether mosquitoes with fewer than six legs survive long enough to engage in host-seeking.

We conclude that loss of up to five legs does not prevent mosquitoes from blood feeding and laying eggs. For this reason, studies of insecticide efficacy should not categorize mosquitoes with fewer than six legs as dead. Leg loss may be an easily observed symptom of pyrethroid exposure, however until its effect upon mosquito fitness in field conditions is quantified, it should not be assumed that leg loss prevents mosquitoes from contributing to disease transmission. The precise classification of mosquitoes as either dead or alive is crucial to studies of insecticide efficacy, as sub-lethal effects are frequently observed. For example, in a study of Permanet 2.0 long-lasting insecticide nets used in Zambia, susceptibility assays revealed that the majority of surviving mosquitoes had 1–3 legs^19^. If both 1- and 2-legged mosquitoes are capable of biting humans, including them in measures of mortality overestimates the efficacy of insecticide-based interventions.

Based on our findings, we suggest that the WHO guidelines for testing of long-lasting insecticidal nets be re-evaluated, as it is possible that mosquitoes with only 1 or 2 legs remain competent as vectors of malaria. A conservative definition of mosquito mortality may serve as a more accurate indicator of insecticide efficacy in vector control. Despite the emergence of insecticide resistance, bed nets and indoor residual spraying remain powerful tools for malaria prevention. The budgets of governments and charities are limited, however, which necessitates strategic deployment of these interventions to regions where their impact upon disease transmission will be greatest. Measures of insecticide efficacy are key to modelling the success of a malaria intervention, and as such must aim to accurately reflect mosquito/insecticide interactions in the field.

## Methods

### Mosquito Rearing and Maintenance

An insecticide susceptible-colony of *An. gambiae* Kisumu strain mosquitoes from western Kenya was used in all experiments. The mosquitoes were maintained at 27 °C with 75% humidity and 12 hour day/night, 1 hour dusk/dawn lighting cycle. Larvae were fed a diet of powdered fish food and adults were provided a 10% sucrose solution *ad libitum*. Adults were maintained in 30 × 30 × 30 cm cages.

### Measurement of blood feeding and egg laying among amputee females

For each of the three experimental replicates, female mosquitoes were drawn haphazardly from a mixed-sex cage of 3–5 day old individuals. This ensured that all females had the same opportunity to mate prior to assignment to treatment groups. Females were divided into three treatment groups, 6 legs (n = 30), 2 legs (n = 45), and 1 leg (n = 30). All females had 6 legs at the start of the experiment. Mosquitoes were anaesthetized on ice and legs were removed by bracing the mosquito thorax with one pair of forceps while pulling the leg with a second pair of forceps. Leg amputation patterns for 1- and 2- legged females were selected to represent all possible leg conformations, which resulted in a higher sample size for the 2-legged group (Supplementary Figure 1). Mosquitoes in the 6-legged group were also anaesthetized on ice. Each cage of mosquitoes (6 legs, 2 legs, 1 leg) was provided with a 10% sucrose-soaked cotton pad on the roof of the cage and a water-soaked cotton pad on the floor of the cage. The following morning, 2–3 hours prior to blood feeding, these cotton pads were removed. Mosquitoes were allowed to blood feed on the hand of a volunteer, which was inserted into the cage for a 6 minute period. The sucrose-soaked cotton was replaced following feeding. Blood feeding success was determined through visual inspection of mosquito abdomens. Two days later, a cup of distilled water lined with filter paper was inserted into each cage, from which all non-blood-fed and dead females had been removed. Two days after placing the egg cup, all eggs were counted using a dissecting microscope and all mosquitoes remaining (the potential mothers) were examined for their leg amputation pattern. The removal of dead and non-blood-fed females prior to cup placement allowed for precise measurement of egg production in living, blood-fed females.

### Measurement of blood feeding and egg laying among insecticide-exposed females

For each of the five experimental replicates, all female mosquitoes were from a mixed-sex cage of 3–5 day old individuals were assayed. Mosquitoes were exposed to standard WHO 0.75% permethrin impregnated papers for 15 minutes utilizing a modified WHO cone test^20^. Preliminary experiments conducted previously had found that exposure to Permanet 2.0 (deltamethrin 1.8 g/kg) caused very high mortality, even with only 1 minute exposure. Mosquitoes were therefore exposed to a standard permethrin impregnated paper for 15 minutes, which generated an adequate number of survivors with leg numbers varying between 0 and 6. A WHO cone test, rather than a WHO tube assay, was conducted because it forces continual contact with the insecticide-treated surface and thus ensured all mosquitoes in the assay receive the same exposure. Batches of ten mosquitoes were introduced into the cone and allowed to contact the insecticide paper for 15 min. Following exposure, mosquitoes were gently removed and placed in recovery cups overnight with access to a 10% sucrose-soaked cotton pad. The following day mosquitoes were classified as dead and alive and sorted into cages based on the number of legs remaining. Replicates ranged in size from 116 to 623 females, with 27 to 213 females surviving exposure. A total of 103 six-legged, 272 two-legged, and 127 one-legged females were assayed for blood feeding and egg laying. The sucrose-soaked cotton pad was withheld for 2–3 hours prior to feeding. 24 hours after exposure, cages of 6, 2 and 1 legged mosquitoes were offered a human hand for 6 minutes. The numbers of blood-fed individuals from each group was recorded and unfed individuals were removed. Three days following blood feeding, single females were placed in 30 ml universal tubes containing wet cotton wool and a disk of filter paper, and allowed to lay eggs. The oviposition rate and the number of eggs obtained for each female was counted 24 hrs subsequently.

### Statistical Analyses

Statistical analyses were conducted using R. Generalized linear mixed model (GLMM) analyses used the *lme4* package. Binary outcomes such as blood feeding or egg laying success were analysed by logistic regression or by mixed-effect logistic regression models with replicate as a random effect. From a Q-Q plot egg batch size was observed to be approximately normal distributed and therefore a linear random effects model was deployed. Ninety-five percent confidence intervals for blood feeding success were calculated by the Newcombe method^21^.

### Use of human subjects

All experiments were performed in accordance with relevant guidelines and regulations, and were approved by the Liverpool School of Tropical Medicine Ethics Review Committee and the Liverpool School of Tropical Medicine Health, Safety, and Environment Management Committee. Informed consent was obtained from all volunteers.

## Additional Information

**How to cite this article:** Isaacs, A. T. *et al*. Insecticide-induced leg loss does not eliminate biting and reproduction in *Anopheles gambiae* mosquitoes. *Sci. Rep.* **7**, 46674; doi: 10.1038/srep46674 (2017).

**Publisher's note:** Springer Nature remains neutral with regard to jurisdictional claims in published maps and institutional affiliations.

## Supplementary Material

Supplementary Video 1

Supplementary Video 2

Supplementary Information
