# Supplementary material for: Insecticide-induced leg loss does not eliminate biting and reproduction in Anopheles gambiae mosquitoes
Source: Sci Rep. 2017 Apr 25;7:46674. doi: 10.1038/srep46674 (PMC5404223; doi:10.1038/srep46674)
Supplement: Supplementary Information [file srep46674-s3.pdf]

Insecticide-induced leg loss does not eliminate biting and reproduction in *Anopheles gambiae* mosquitoes

Alison T. Isaacs, Amy Lynd, Martin J. Donnelly

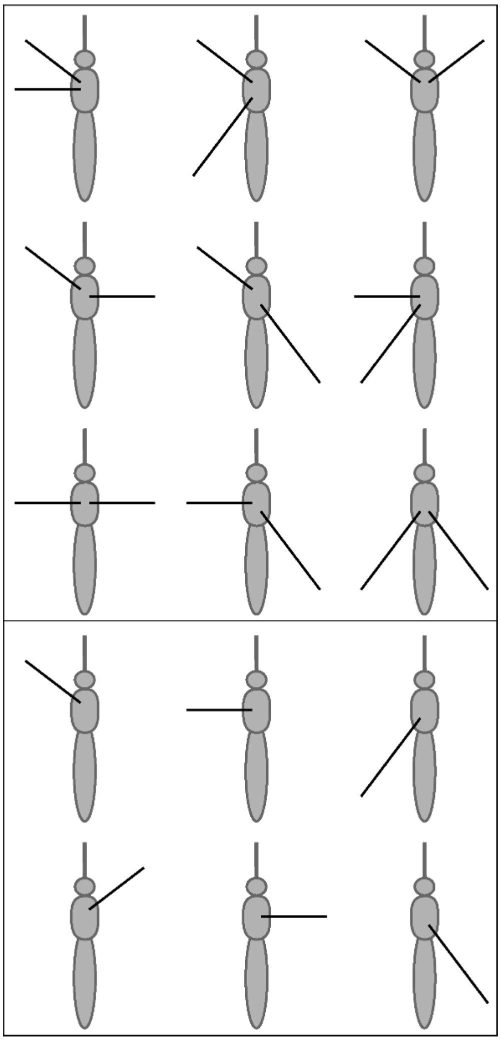

Supplementary Figure 1. A ventral view of leg amputation patterns for 2-legged mosquitoes (top), and 1-legged mosquitoes (bottom).

Supplementary Video 1. Video of mosquito behaviour following exposure to an untreated bed net, a Permanet 2.0 bed net (deltamethrin-impregnated), or an Olyset bed net (permethrin-impregnated) in a WHO cone bioassay. Mosquitoes were observed at two timepoints: within the first hour post-exposure and 3 hours post-exposure.

Supplementary Video 2. Video of 6-, 2-, and 1-legged mosquito blood feeding behaviour.
